# Usefulness of a small-caliber tip transparent hood in endoscopic submucosal dissection for pharyngeal cancer

**DOI:** 10.1055/a-2408-9885

**Published:** 2024-09-19

**Authors:** Yugo Suzuki, Yorinari Ochiai, Sena Eda, Minoru Oda, Tsuyoshi Ishii, Shu Hoteya

**Affiliations:** 1 Department of Gastroenterology, Toranomon Hospital, Tokyo, Japan

Endoscopic submucosal dissection (ESD) for superficial pharyngeal cancer is becoming an established minimally invasive treatment with favorable short- and long-term outcomes 1 2 3 . In recent years, a small-caliber tip transparent hood (CAST hood; TOP, Tokyo, Japan) has been useful in cases involving severe fibrosis and pocket creation 4 .

A 69-year-old man with a history of ESD for esophageal cancer was referred to our hospital for an erythematous lesion on the posterior wall of the hypopharynx detected by upper gastrointestinal endoscopy during a routine examination ( Fig. 1 **a–d** ). A tissue biopsy confirmed the diagnosis of squamous cell carcinoma, and we performed ESD ( Video 1 ) under general anesthesia using a GIF-H290T (Olympus, Tokyo, Japan) and DualKnife J (KD-655Q; Olympus). An incision was made on the anal side using a transparent distal attachment hood (D-201-11804; Olympus). A circumferential incision was then made, and the distal attachment was changed to the CAST hood. The lesion was resected en bloc within 24 minutes without the use of traction devices and any adverse events ( Fig. 2 **a–i** ). Histopathology confirmed R0 resection of a 14 × 12-mm large well-differentiated squamous cell carcinoma that had invaded the subepithelium with a tumor thickness of 350 μm ( Fig. 3 **a, b** ).

**Fig. 1:**
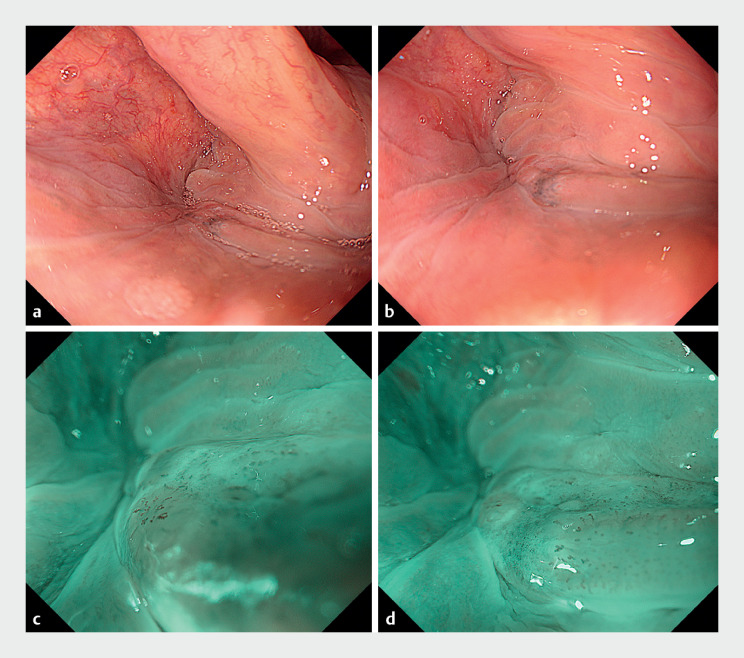
Pretreatment endoscopic evaluation. **a, b** An erythematous 0-IIb lesion with partial melanosis was seen at the posterior wall of the hypopharynx. The distal side of the lesion was not fully visible due to the postcricoid region riding over it. **c, d** Magnified endoscopy with narrow-band imaging showing a brownish lesion with dilated and irregularly aligned intrapapillary capillary loops, suggesting that tumor invasion was limited to the subepithelial layer.

**Fig. 2:**
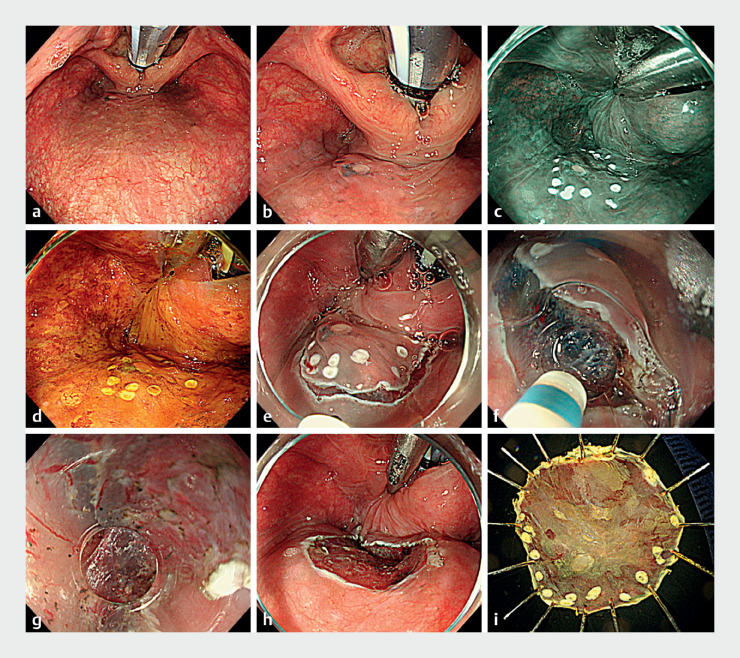
Endoscopic procedure. **a, b** A curved rigid laryngoscope was used to expand the larynx to visualize the entire lesion. **c** Markings were made 5 mm outside the lesion while lifting the postcricoid region with laryngeal forceps to ensure visibility. **d** Lugol’s iodine staining shows a Lugol-voiding area consistent with the brownish area of narrow-band imaging. **e** An incision was made from the anal side, followed by a circumferential incision. **f, g** The small-caliber tip transparent hood facilitated visualization and dissection of the submucosal layer through a shallow incision even in a confined space, making it easier to get under the specimen. **h, i** The lesion was resected en bloc without any adverse events.

**Fig. 3:**
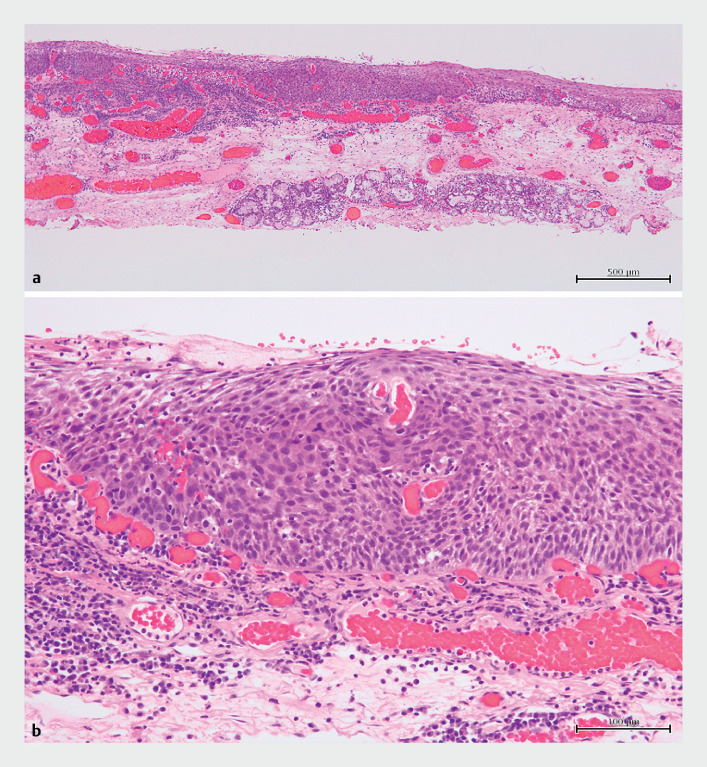
Histological examination findings of the resected specimen. **a, b** Photomicrographs ([ **a** ] × 40, [ **b** ] × 200) of the lesion with hematoxylin and eosin staining. Tumor cells infiltrating the subepithelial layer without lymphovascular invasion.

Successful endoscopic submucosal dissection of pharyngeal cancer using a small-caliber tip transparent hood.Video 1

ESD for pharyngeal carcinoma can be difficult because of the limited working space. While the ultrathin endoscope has highly flexible operability 5 , it does not have a water jet function, and suction cannot be used during treatment. The CAST hood is a transparent hood with a tip diameter of 4 mm, making it easier to maintain visibility and perform fine manipulation in confined spaces. The gradual increase in diameter from the tip allows natural traction, minimizing the amount of glycerol injected and the risk of laryngeal edema. These findings suggest that the CAST hood is particularly suitable for the endoscopic treatment of pharyngeal cancer lesions.

Endoscopy_UCTN_Code_TTT_1AO_2AG_3AD

## Notes

Endoscopy E-Videos https://eref.thieme.de/e-videos
*E-Videos* is an open access online section of the journal *Endoscopy* , reporting on interesting cases and new techniques in gastroenterological endoscopy. All papers include a high-quality video and are published with a Creative Commons CC-BY license. Endoscopy E-Videos qualify for HINARI discounts and waivers and eligibility is automatically checked during the submission process. We grant 100% waivers to articles whose corresponding authors are based in Group A countries and 50% waivers to those who are based in Group B countries as classified by Research4Life (see: https://www.research4life.org/access/eligibility/ ). This section has its own submission website at https://mc.manuscriptcentral.com/e-videos .

## References

[1] Iizuka T Kikuchi D Hoteya S Endoscopic submucosal dissection for treatment of mesopharyngeal and hypopharyngeal carcinomas Endoscopy 2009 41 113 117 10.1055/s-0028-1119453 19214888

[2] Shimizu Y Yamamoto J Kato M Endoscopic submucosal dissection for treatment of early stage hypopharyngeal carcinoma Gastrointest Endosc 2006 64 255 259 10.1016/j.gie.2006.01.049 16860078

[3] Iizuka T Kikuchi D Suzuki Y Clinical relevance of endoscopic treatment for superficial pharyngeal cancer: feasibility of techniques corresponding to each location and long-term outcomes Gastrointest Endosc 2021 93 818 827 32721490 10.1016/j.gie.2020.07.039

[4] Kikuchi D Tanaka M Suzuki Y Endoscopic submucosal dissection for superficial pharyngeal carcinoma using transnasal endoscope VideoGIE 2021 6 67 70 10.1016/j.vgie.2020.10.004 33884330 PMC7859500

[5] Nomura T Sugimoto S Oyamada J GI endoscopic submucosal dissection using a calibrated, small-caliber-tip, transparent hood for lesions with fibrosis VideoGIE 2021 6 301 304 34278091 10.1016/j.vgie.2021.03.001 PMC8267961

